# Do payers value rarity? An analysis of the relationship between disease rarity and orphan drug prices in Europe

**DOI:** 10.1080/20016689.2017.1299665

**Published:** 2017-04-10

**Authors:** Goran Medic, Daria Korchagina, Katherine Eve Young, Mondher Toumi, Maarten Jacobus Postma, Micheline Wille, Michiel Hemels

**Affiliations:** ^a^ Horizon Pharma Europe B.V., Market Access EMEA, AH Utrecht, the Netherlands; ^b^ Unit of Pharmacoepidemiology and Pharmacoeconomics, Department of Pharmacy, University of Groningen, Groningen, the Netherlands; ^c^ Mental Health and Public Health, University Paris-Sud, Maison de Solenn, Paris, France; ^d^ Creativ-Ceutical, Paris, France; ^e^ Public Health Department, Research Unit, Aix-Marseille University, Marseille, France

**Keywords:** Rare diseases, orphan drugs, pricing, treatment cost, Europe

## Abstract

**Background and Objective**: Orphan drugs have been a highlight of discussions due to their higher prices than non-orphan drugs. There is currently no European consensus on the method of value assessment for orphan drugs. This study assessed the relationship between the prevalence of rare diseases and the annual treatment cost of orphan drugs in France, Germany, Italy, Norway, Spain, Sweden, and UK.

**Methods**: Approved orphan drugs and prevalence data were extracted from the European Medicines Agency website. Annual treatment costs were calculated using ex-factory price. Simple regression was used to analyse the relationship between costs and prevalence. A specific bivariate analysis was performed for the rarest diseases (≤1 per 10,000).

**Results**: 120 drugs were analysed. Prevalence ranged from 0.001 to 5 per 10,000 (mean 1.24, median 1). Annual treatment costs per patient ranged from €755 to €1,051,956 (mean €100,000, median €39,303). Results show a statistically significant inverse correlation between annual treatment cost and disease prevalence in all countries (France: r = −0.370, *p *= 0.002; Germany: r = −0.365, *p *= 0.002; Italy: r = −0.340, *p *= 0.002; Spain: r = −0.316, *p *= 0.041; UK: r = −0.358, *p *= 0.0004; Sweden: r = −0.414, *p *= 0.014; Norway: r = −0.367, *p *= 0.002). When analysis was focused on the rarest diseases, a stronger correlation exists in all countries (France: r = −0.525, Germany: r = −0.482, Italy: r = −0.497, Spain: r = −0.531, UK: r = −0.436, Sweden: r = −0.455, Norway: r = −0.466; all *p *< 0.05 except Sweden *p *= 0.077).

**Conclusions**: This study shows an inverse correlation between annual treatment cost and prevalence with high statistical significance in the studied countries. Although pricing is a complex process where different attributes are assessed, this study supports the idea that payers value rarity in pricing decisions.

## Introduction

### Rare disease definition and burden

Rare diseases are uncommon and serious conditions which are defined in the European Union (EU) as life-threatening or chronically debilitating conditions with a prevalence of no more than five in 10,000 people.[1] In the UK, the National Institute of Health and Clinical Excellence (NICE) in England and Wales has defined ultra-rare diseases as diseases affecting less than 1000 patients in the UK.[2] Orphan drugs are medicines intended for the diagnosis, prevention or treatment of rare diseases.

Rare diseases are usually severe conditions with no or limited choice of therapeutic options, and thus present with a high level of unmet need. In 2007, the EMA estimated that there are 5000 to 8000 rare diseases affecting between 6% to 8% of the total EU population, amounting to 27 million to 36 million people in the EU.[1] The same report documented that five new diseases are described in the medical literature every week, hence current figures are assumed to be higher. It is estimated that only 1% are currently covered by approved treatments in the EU.[3]

### Rare disease legislation and developments

Prior to 2000, commercial drug development in rare diseases was prohibitive and limited. Due to the very small number of patients affected, manufacturers have been reluctant to invest in the research and development (R&D) of orphan drugs as return on investment is improbable. In 2000, EU legislation 141/2000,[4] also known as the European Orphan Medicinal Products Regulation, has established a centralised procedure for the designation of orphan medicinal products and has put in place regulatory and economic incentives for the research, marketing, and development of orphan drugs. It has allowed the review and approval of orphan drugs, upholding the principle of equity in patient access to treatment which underpins the legislation.

The orphan drug legislation in Europe has been considered a success since its enactment. As of July 2016 and more than 15 years after its inception, the European Commission has designated 1329 products as orphan medicinal products and has authorised 126 orphan medicines for the benefit of patients suffering from rare diseases.[5] Orphan drug designation and marketing authorisation are two different procedures set in continuum, both under the remit of the European Medicines Agency (EMA). Although an orphan designation may be granted once the criteria of disease severity, low prevalence, and the lack of satisfactory treatment options have been fulfilled, marketing authorisation approval has regulatory requirements of safety and efficacy that must be met. The incentives provided by the orphan drug legislation aim to incentivise and support manufacturers through drug development and accelerate regulatory assessment. However, many products that are eligible for orphan designation may not be commercially viable or may not be funded for development. Some products are based on an off-patent commercially available molecule and competition with potential generics may prevent return on investment. Some products may be based on a patent-protected product and may need to wait for the product to become off patent to initiate development. Orphan designation is a relatively easy process and does not require significant investment, while drug development to achieve marketing authorisation requires a very high investment, and is very risky as success rates for development are relatively low. Manufacturers may opt not go through the marketing authorisation process if the clinical proofs of concept are negative. Nonetheless, the orphan drug legislation has seen an increase in marketing authorisations in rare diseases. The EMA has recommended the highest number of orphan designated medicines for marketing authorisation in a year in 2015: 18 approvals were intended for rare disease compared to 40 approvals for non-orphan new medicinal products. This is an increase from the 17 orphan drug approvals in 2014, and the 11 drug approvals in 2013.[6]

### The high costs of orphan drugs

The success which has resulted in an increasing number of licensed medicines for rare diseases has also resulted to a growing debate that relates to high costs and affordability in the wake of the continuing economic crisis and health budget austerity measures in Europe.[7–14]

Orphan drugs have been a highlight of discussions due to their higher price than non-orphan drugs. A 2011 budget impact study in 18 countries in Europe [7] showed that the annual patient cost of commercially available orphan drugs varied between €1,251 and €407,631 with a median cost of €32,242 per treatment year per patient. The share of the total pharmaceutical market represented by orphan drugs was predicted to peak from 3.3% in 2010 to 4.6% in 2016, and plateau at 4–5% until 2020, where absolute expenditure will increase, but no faster than the growth of the greater EU pharmaceutical market.[7]

### Pricing of orphan drugs and surrounding issues

Price setting is a multifactorial decision with various determinants such as R&D risk, return on investment, unmet needs, availability of alternative therapies, the drug’s additional value to current treatment options, incremental cost effectiveness ratio, and pricing and reimbursement (P&R) policies and processes. Although orphan drugs are subjected to the usual pharmaceutical drug pricing rationale in most countries, the European legislation incentives have directly and indirectly influenced the P&R landscape. In the literature, this has been referred to as the ‘special market access status’ of orphan drugs.[11] Orphan drugs’ small target population has resulted in a need for high prices, so that manufacturers have the return on investment to cover costs for drug development and post-marketing authorisation surveillance. The high prices also create an incentive for manufacturers to pursue investment in the development of orphan drugs. As the costs and margins have to be recovered from a limited number of patients, this has led to high costs per patient.[12,15] Second, as they often are the only available treatment option, some orphan drugs are considered to have high value and thus benefit from high prices.[12] A study by Picavet et al. [13] found that designated orphan drugs achieve a higher median price than non-designated orphan drugs that also target rare diseases (*p *< 0.01). Third, 10 years of market exclusivity has led to monopolistic situations where manufacturers are in a position of power to set prices.[8,11,12] Lastly, payers tend to face pressure from patient groups regarding access to medicines and thus comply to manufacturers’ price demands as the rationale underpinning the EU legislation is equity in access to treatment.[12]

Price [8] and access of orphan drugs vary among countries in the EU.[16–19] Although orphan designation and marketing authorisation is at a European level, pricing and reimbursement are on a national level often driven by health technology assessments (HTA) outcomes and a variable impact from external reference pricing.[20] In these HTAs, evidence requirements, pricing and reimbursement decision frameworks, and budget ceilings vary. Thus, prices and levels of access vary.

National pricing regulations are often value-based and the value placed on orphan drugs, as with any intervention, varies per health care system. How much they are willing to pay for a certain value is also a relevant differentiator among countries. Some may value equity where all patients deserve treatment and put precedence on products that treat the greatest health need, regardless of the high budget impact of the orphan drug, while some may value maximising health outcomes in the face of budget constraints.[18] Often, it will be a combination of these arguments. Other value drivers may include disease rarity, disease severity, the availability of treatment options, the size of clinical benefit, and incremental cost-effectiveness ratio. Drug budget impact is rarely considered despite the high per-patient price, due to low patient numbers in rare diseases, and thus the drug budget impact is usually low. In the UK, a societal preference survey [2] done by NICE in 2004 showed that disease severity, the size of the clinical benefit, and life threatening disease may be valued in the NHS for orphan drug funding. Disease rarity was not a reason found significant to pay for price premium.[2] This matches well with the British health technology evaluation culture which puts efficiency first in economic evaluation by maximising the total health gain through cost per quality-adjusted life years (QALY) computations. QALY, the measure of health gain, is the outcome used to assess the appropriate use of limited health care resources. The UK seems to consider a QALY is a QALY, regardless of who gains or loses it, and the willingness to pay is not driven by rarity.[21] Exceptions however exist for end-of-life treatments with QALYs reflecting quantitative assessment and rarity and equity considered from a qualitative and societal point of view.

It has been questioned whether the current value assessment frameworks truly reflect social preferences and values in terms of funding orphan drugs.[21] The UK study described above showed no explicit societal values prioritising rarity.[2] In a Norwegian survey of 1479 people, 80% answered that rare diseases should have the same equal access to health care regardless of costs but only 42% were willing to equally divide health care funds between rare and common diseases.[22] Although most studies such as these are small and have been suggested to have methodological flaws and thus should be interpreted with caution, Drummond et al. [9] have discussed that these conflicting findings may be explained by two notions of equity: horizontal equity versus vertical equity. Horizontal equity is defined as the equal treatment of equals.[9] On the other hand, vertical equity is the unequal but equitable treatment of unequals.[9,21] A health care system which uses a single cost per QALY threshold for all reflects horizontal equity. A health care system which regards the unique state of patients with rare diseases and that these patients are equally entitled to treatment even if it means foregoing efficiency standards reflects vertical equity. As to which should be prioritised is still an ongoing discussion and the answer may differ per institution.[9]

As a fair amount of literature has argued on how orphan drugs should be treated in terms of pricing and market access,[8,9,11,23] collaborations have been proposed to better understand the value of orphan drugs in light of P&R decisions.[21] As the usual HTA frameworks have been criticised to be limited for the complete evaluation of orphan drugs,[9,15,24] multiple criteria decision analysis (MCDA) frameworks which incorporate relevant value elements into P&R decision in a transparent and consistent matter [25–28] and other price control mechanisms such as cost-plus or rate of return models employing yardsticked cost allocations and rate of return calculations in setting orphan drug prices [29] have been proposed.

### Significance and objective of the study

Studies on how orphan drugs are priced in Europe are sparse and the pricing of these drugs has been referred to as a black box.[12,14] There is currently no European consensus on how the value of orphan drugs are and should be assessed.[24]

With the ongoing discussion on should we value rarity and on how European health care systems should assess and price orphan drugs, understanding the value drivers that payers attach to orphan drugs is important. Studies focusing on evaluating the present system will pave the way to new pricing and reimbursement strategies.

As prevalence is the cornerstone of orphan drug designation, do payers value rarity in pricing decisions? Do payers seem to accept higher prices for orphan drugs, as initially priced by manufacturers? The objective of this study is to assess the relationship between the annual cost of treatment per patient of orphan drugs (price) and the prevalence (rarity) of the corresponding rare diseases in Europe.

## Methodology

A five-step process was implemented in order to assess the relationship between the annual cost of treatment of orphan drugs and the prevalence of the corresponding rare diseases: (1) extraction of the approved orphan drugs from the European Medicines Agency (EMA) website; (2) extraction of ex-factory price for all products in the countries of scope from IHS POLI database and country-specific price database; (3) calculation of annual treatment cost per patient; (4) extraction of disease prevalence at EU level from the EMA website; and (5) analysis of annual treatment costs versus disease prevalence. The countries included were France, Germany, Italy, Norway, Spain, Sweden and the UK.

### Extraction of orphan drugs from EMA website

We searched the EMA database for the list of approved orphan drugs and their approved indications. Orphan drugs granted marketing authorisation up to 13 June 2016 including drugs with expired or withdrawn orphan drug designations were extracted for analysis. Only one indication per orphan drug was used in the analysis. If the orphan drug was approved for more than one indication, the first EMA indication approved was chosen for inclusion in the analysis. If both indications were approved at the same time, the least prevalent indication was chosen for inclusion.

### Extraction of ex-factory price from IHS POLI database and country-specific price database

The IHS POLI database [30] was the primary source of price data. For drugs with withdrawn and expired orphan designations with no available prices in POLI, available country-specific price databases were used: Database of drugs and tariffs (Ameli) [31] for France, British National Formulary (BNF) [32] for the UK, and Farmadati Compendio Farmaceutico Telematico database [33] for Italy.

The earliest price was used for cost calculation as we are interested in the prices at launch and drug prices change over time. An exception to this was when using BNF, where current prices were extracted because price history was not available.

Prices in British pound sterling, Swedish krona, and Norwegian krone, were converted to Euros by applying the respective exchange rates: €1 = £0.72, €1 = 9.09 Swedish Krona, €1 = 9.09 Norwegian Krone. Conversion was done by the IHS database system upon extraction and the same conversion rates were used for BNF prices.

### Calculation of annual treatment cost per patient

We calculated the annual treatment cost per patient in each country for each orphan drug based on the annual treatment dose according to the standard treatment plan described in the Summary of Product Characteristics (SmPC). Across all seven countries, the indication and posology are the same. There are differences in the preparation and formulation of drugs across countries but these are minor. As much as possible, the same formulation and preparation per product were used in all countries for ease of comparability.

Assumptions were used during dose and cost computations as dosing of orphan drug treatments may vary according to patient age, weight, disease severity, patient needs, disease progression, or disease complications.

- Average drug dose for an adult was used unless the drug would be specifically indicated for use in children. For drugs indicated for both adults and paediatric populations, the pivotal studies described in the European Public Assessment Report (EPAR) were consulted for the average age range of the population included in clinical trials and dosage and cost computation were done for this specific average patient. For weight adjusted and body surface area (BSA) adjusted treatments, the average weight of an adult is set at 70 kg and the average body surface area is set at 1.73 m^2^. Standard average values for other age intervals were also used.[34]
- If the dose is adjustable based on performance results or an average dose was given, information regarding the average treatment duration and dosage from the EPAR and pivotal studies were used. In the same manner, for cycle-based treatments where the number of cycles varies, the mean number of cycles in the pivotal trials was assumed.
- Treatment duration of 365 days was assumed. For drugs used for less than a year, the costs of the total treatment course were analysed as annual costs.
- For treatments administered as injection or infusion, the nearest full vial size was used. The EPAR was consulted if vials can be stored once opened or should be used within the day. Vial wastage in this sense was taken into consideration.
- If there was an unfinished pack at the end of the year or at the end of a treatment cycle, only a proportion of the price of that pack was accounted for.

### Extraction of disease prevalence at EU level from the EMA website

The prevalence of rare diseases reported in the EMA website [35] were used for analysis. The reported prevalence rates were at the EU level thus the same rare disease prevalence was used for all country analysis. If the prevalence was reported as a range, the average prevalence was used. If the prevalence was indicated as less than a certain number, the nearest number less than the indicated number was used (e.g. less than 0.2 per 10,000 is 0.19).

### Analysis of annual treatment costs versus disease prevalence

Straightforward linear regression analysis was done and correlation coefficients were computed to determine the relationship between the annual treatment cost and prevalence of rare diseases per country. Results were plotted per country. A significant number of orphan drugs were for the rarest diseases (prevalence 0–1 per 10,000) and a specific bivariate analysis between annual costs and prevalence was performed for this very low-prevalence cohort. A specific linear regression was also done for France referencing the ASMR (Amélioration du Service Médical Rendu) scores for each orphan drug from the health technology assessments (HTA) done by Haute Autorité de Santé (HAS). ASMR is a driver of price setting in France. Thus, the sub-analysis assessed the relationship between disease prevalence and ASMR, and ASMR and annual treatment costs.

## Results

Ninety-five authorised orphan drugs were extracted from the EMA website and were complemented with 25 drugs with expired or withdrawn orphan drug designations, for a total of 120 (Table 1). The prevalence ranged from 0.001 to 5 patients per 10,000 with a mean of 1.24 per 10,000 and a median of 1 per 10,000.

**Table 1.** Drugs used in the analysis with corresponding indication and prevalence.

Drug	Indication simplified	Prevalence per 10,000
Adcetris	Hodgkin disease	1.00
Adempas	Pulmonary hypertension	2.00
Afinitor*	Renal cell carcinoma	4.20
Aldurazyme*	Mucopolysaccharidosis I	0.03
Alprolix**	Haemophilia B	0.20
Arzerra	Chronic lymphocytic leukaemia	3.50
Atriance	Precursor T-Cell lymphoblastic leukaemia-lymphoma	1.10
Blincyto	Precursor cell lymphoblastic leukaemia-lymphoma	1.00
Bosulif	Chronic myeloid leukaemia	1.60
Bronchitol	Cystic fibrosis	1.30
Busilvex*	Conditioning treatment prior to haematopoietic-progenitor-cell transplantation	0.70
Carbaglu	N-acetylglutamate synthetase deficiency	0.00
Cayston	Gram negative bacterial lung infection in cystic fibrosis	1.30
Ceplene	Acute myeloid leukaemia	0.70
Cerdelga	Gaucher disease type 1	0.30
Coagadex**	Hereditary factor X deficiency	0.09
Cometriq	Metastatic medullary thyroid carcinoma	0.70
Cresemba	Mucormycosis	0.06
Cyramza*	Stomach neoplasms (Gastric cancer)	3.00
Cystadane	Homocystinuria	0.17
Dacogen	Acute myeloid leukaemia	1.00
Darzalex**	Plasma cell myeloma	1.75
Defitelio**	Severe hepatic veno-occlusive disease	0.40
Deltyba	Tuberculosis	2.00
Diacomit	Severe myoclonic epilepsy in infancy	0.40
Elaprase	Mucopolysaccharidosis II	0.02
Esbriet	Idiopathic pulmonary fibrosis	3.00
Evoltra	Precursor cell lymphoblastic leukaemia-lymphoma	0.40
Exjade	Iron overload	2.70
Fabrazyme*	Fabry disease	0.03
Farydak	Multiple myeloma	3.20
Firazyr	Hereditary angioedemas	2.50
Firdapse	Lambert-Eaton myasthenic syndrome	0.10
Galafold**	Fabry disease	1.00
Gazyvaro	Chronic lymphocytic leukaemia (follicular lymphoma)	2.40
Gliolan	Malignant glioma	3.70
Glivec*	Chronic myeloid leukaemia	0.90
Glybera	Familial lipoprotein lipase deficiency	0.02
Granupas	Tuberculosis	2.00
Hetlioz**	Non-24-hour sleep-wake disorder	1.85
Holoclar**	Limbal stem cell deficiency	0.30
Iclusig	Chronic myeloid leukaemia	0.80
Idelvion	Haemophilia B	0.10
Ilaris*	Cryopirin associated syndromes	0.05
Imbruvica	Mantle cell lymphoma	0.17
Imnovid	Multiple myeloma	2.20
Increlex	Laron syndrome	2.00
Inovelon	Epilepsy	1.50
Jakavi*	Chronic idiopathic myelofibrosis	0.50
Kalydeco	Cystic fibrosis	1.20
Kanuma	Lysosomal acid lipase deficiency	0.20
Ketoconazole HRA	Endogenous Cushing’s syndrome	0.90
Kolbam	Inborn errors in primary bile acid synthesis	0.07
Kuvan	Phenylketonurias	1.70
Kyprolis	Multiple myeloma	1.30
Lenvima	Differentiated thyroid carcinoma	0.60
Litak*	Hairy cell leukaemia	2.40
Lynparza	Ovarian cancer	2.90
Lysodren*	Adrenal cortex neoplasms	0.10
Mepact	Osteosarcoma	0.50
Mozobil	Hematopoietic stem cell transplantation for multiple myeloma, lymphoma	1.00
Myozyme	Glycogen storage disease type II	0.14
Naglazyme*	Mucopolysaccharidosis VI	0.02
Nexavar	Renal cell carcinoma	3.01
Nexobrid	Deep partial- and full-thickness thermal burns	1.00
Nplate	Thrombocytopenic idiopathic purpura	1.00
Ofev	Idiopathic pulmonary fibrosis	3.00
Opsumit	Pulmonary arterial hypertension	1.80
Orfadin*	Tyrosinemias	0.10
Orphacol	Digestive system diseases, inborn errors of metabolism	0.06
Pedea*	Patent ductus arteriosus	2.13
Peyona	Primary apnoea	0.85
Photobarr*	Barrett oesophagus	3.60
Plenadren	Adrenal insufficiency	4.50
Prialt*	Pain injections, spinal	1.54
Procysbi	Nephropathic cystinosis	0.10
Raxone	Leber’s hereditary optic neuropathy	1.00
Ravicti**	Carbamoyl-phosphate synthase-1 deficiency	0.14
Replagal*	Fabry disease	0.02
Revatio*	Pulmonary hypertension	1.00
Revestive	Short-bowel syndrome	0.20
Revlimid	Multiple myeloma	1.30
Revolade*	Idiopathic thrombocytopenic purpura	2.50
Savene	Extravasation of diagnostic and therapeutic materials	0.03
Scenesse**	Erythropoietic protoporphyria	0.19
Signifor	Cushing’s disease	1.20
Siklos	Sickle cell anaemia	0.50
Sirturo	Pulmonary multidrug resistant tuberculosis	2.00
Soliris	Paroxysmal nocturnal haemoglobinuria	0.10
Somavert	Acromegaly	0.60
Sprycel	Chronic myelogenous leukaemia, BCR-ABL positive	0.89
Strensiq	Hypophosphatasia	0.10
Strimvelis	Adenosine deaminase deficiency	0.02
Sutent*	Malignant gastrointestinal stromal tumours	0.30
Sylvant	Multicentric Castlemans disease who are human immunodeficiency virus (HIV) negative and human herpesvirus-8 (HHV-8) negative	0.99
Tasigna	Chronic myelogenous leukaemia, BCR-ABL positive	1.00
Tepadina	Conditioning treatment prior to allogeneic or autologous haematopoietic progenitor cell transplantation (HPCT) in haematological diseases	0.60
Thalidomide-Celgene	Multiple myeloma	1.20
Thelin	Pulmonary arterial hypertension	0.50
Tobi Podhaler	Pseudomonas aeruginosa lung infection in cystic fibrosis	1.30
Torisel	Renal cell carcinoma	3.50
Tracleer*	Pulmonary arterial hypertension and chronic thromboembolic pulmonary hypertension	0.95
Translarna**	Duchenne muscular dystrophy	0.30
Trisenox*	Acute promyelocytic leukaemia	0.80
Unituxin**	Neuroblastoma	1.10
Uptravi	Pulmonary arterial hypertension	1.80
Ventavis*	Primary pulmonary hypertension	2.20
Vidaza	Myelodysplastic syndromes	2.05
Vimizim	Mucopolysaccharidosis, type IVA (Morquio A syndrome)	1.40
Volibris	Pulmonary arterial hypertension and chronic thromboembolic pulmonary hypertension	2.00
Votubia	Renal angiomyolipoma with tuberous sclerosis complex	1.00
VPRIV	Gaucher disease	0.30
Vyndaqel	Transthyretin amyloidosis	2.90
Wakix**	Narcolepsy	5.00
Wilzin*	Hepatolenticular degeneration	0.60
Xagrid	Essential thrombocythaemia	2.50
Xaluprine	Acute lymphoblastic leukaemia	1.20
Xyrem*	Narcolepsy	5.00
Yondelis	Sarcoma	0.61
Zavesca	Gaucher disease	0.60

*Orphan designation withdrawn or expired.

** No prices available in the countries in scope; probably not commercially available.

Not all orphan drugs are commercially available in all seven countries and not all commercially available orphan drugs had available prices for analysis. For example, in many countries, drugs for hospital use do not have listed prices. The prices for these drugs are negotiated on a case by case basis with each hospital and are confidential. The number of drugs analysed per country is presented in Table 2.

**Table 2.** Number of orphan drugs analysed per country.

Country	Number of orphan drugs with prevalence of 0–5 per 10,000	Number of orphan drugs with prevalence of 0–1 per 10,000
France	67	36
Germany	68	36
Italy	83	45
Norway	67	36
Spain	42	19
Sweden	35	16
UK	94	52

**Figure 1.**
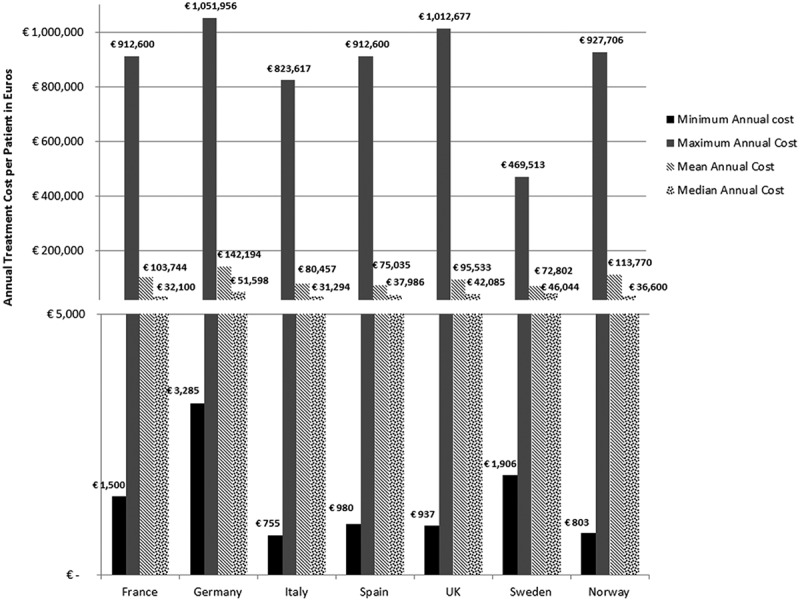
Orphan drugs annual treatment costs in seven EU countries.

In all seven countries, the annual treatment costs per patient ranged from €755 to €1,051,956 with a mean of €100,000 and median of €39,303. Country differences in annual treatment costs are shown in Figure 1. Germany had the highest mean annual treatment cost, followed by Norway, France, UK, Italy, Spain, and Sweden, respectively.

In all the countries, results showed an inverse correlation between disease prevalence and annual treatment cost with the rarer the disease, the more expensive the treatment (FR: r = −0.370, *p *= 0.002; DE: r = −0.365, *p *= 0.002; IT: r = −0.340, *p *= 0.002; ES: r = −0.316, *p *= 0.041; UK: r = −0.358, *p *= 0.0004; SE: r = −0.414, *p *= 0.014; NO: r = −0.367, *p *= 0.002). Very high statistical significance was met in all countries.

Of the drugs, 53% were in the very low prevalence cohort (prevalence 0–1 per 10,000) and when analysis was focused on these rarest diseases, a stronger correlation was found in all countries (FR: r = −0.525, *p *= 0.001; DE: r = −0.482, *p *= 0.003; IT: r = −0.497, *p *= 0.001; ES: r = −0.531, *p *= 0.019; UK: r = −0.436, *p *= 0.001; SE: r = −0.455, *p *= 0.077; NO: r = −0.466, *p *= 0.004; all *p *< 0.05 except SE) (Figures 2–15).

**Figure 2.**
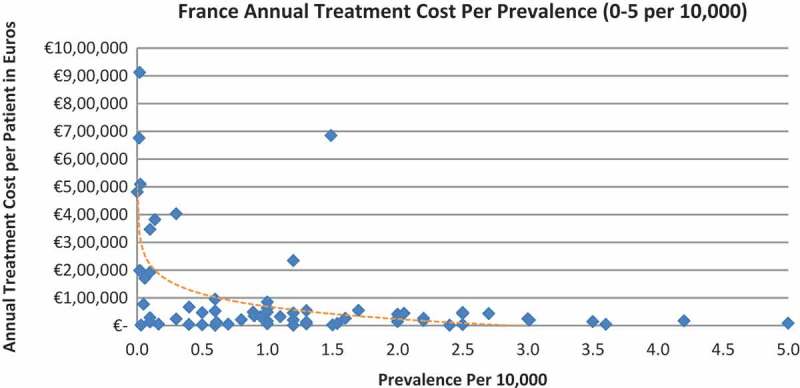
France annual treatment cost per prevalence (0–5 per 10,000).

**Figure 3.**
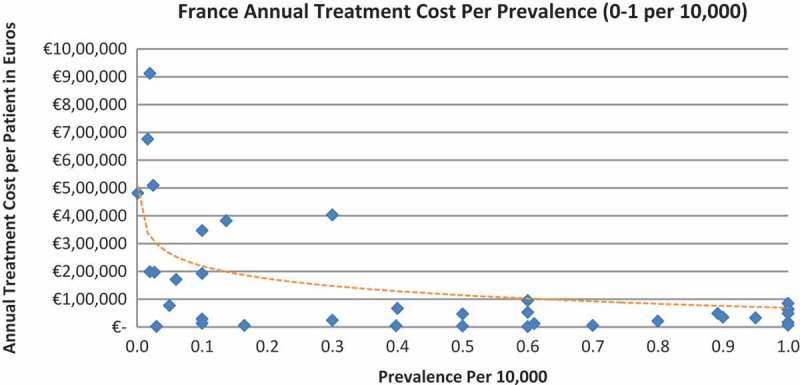
France annual treatment cost per prevalence (0–1 per 10,000).

**Figure 4.**
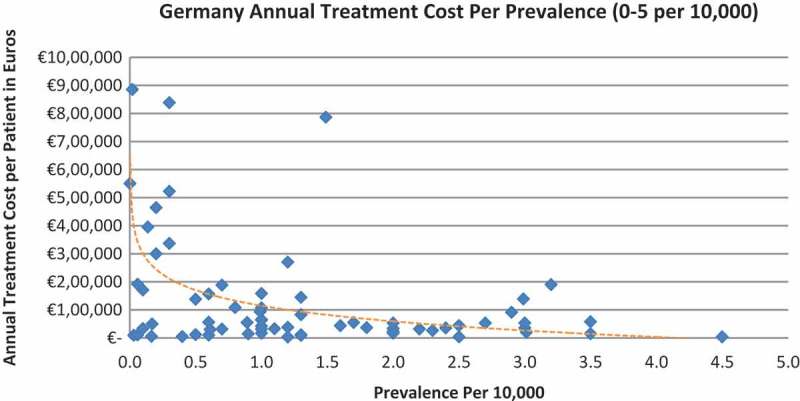
Germany annual treatment cost per prevalence (0–5 per 10,000).

**Figure 5.**
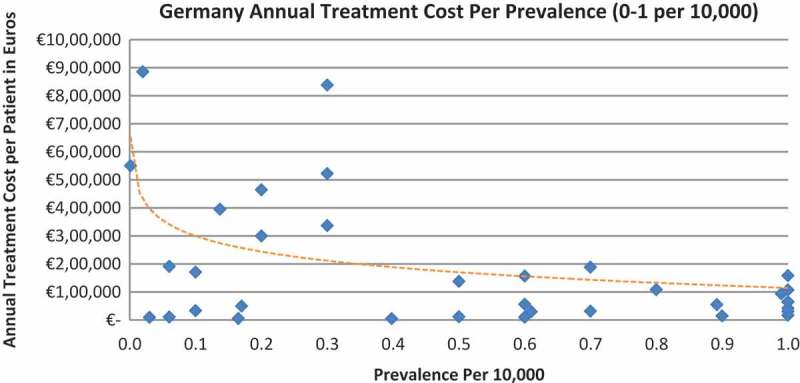
Germany annual treatment cost per prevalence (0–1 per 10,000).

**Figure 6.**
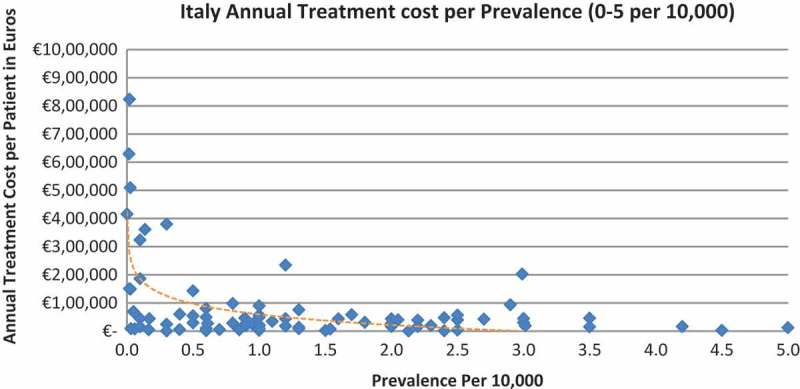
Italy annual treatment cost per prevalence (0–5 per 10,000).

**Figure 7.**
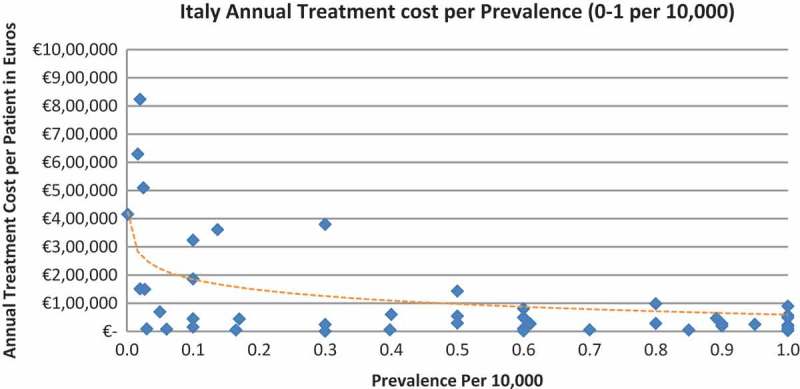
Italy annual treatment cost per prevalence (0–1 per 10,000).

**Figure 8.**
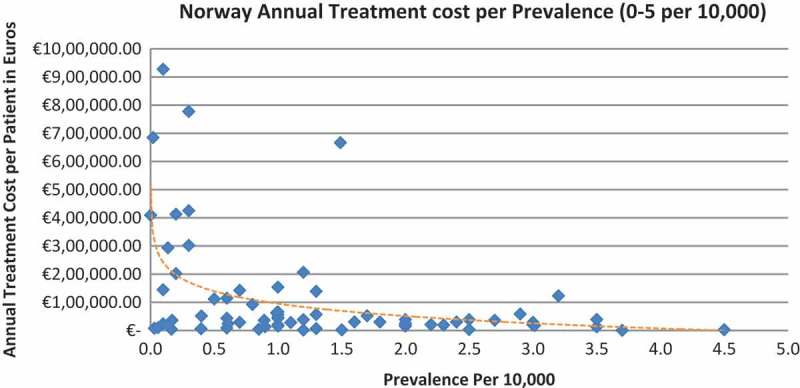
Norway annual treatment cost per prevalence (0–5 per 10,000).

**Figure 9.**
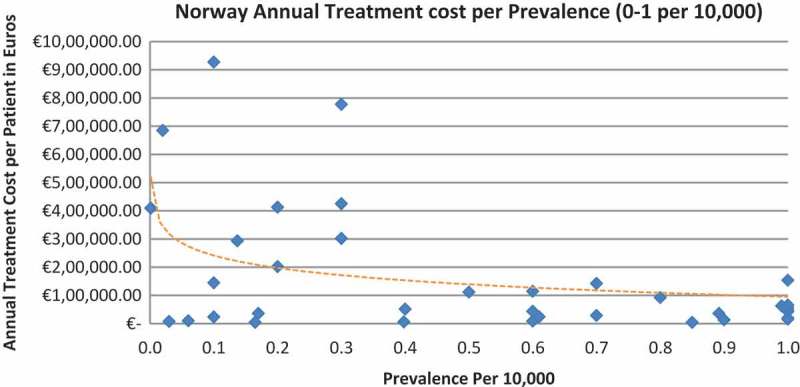
Norway annual treatment cost per prevalence (0–1 per 10,000).

**Figure 10.**
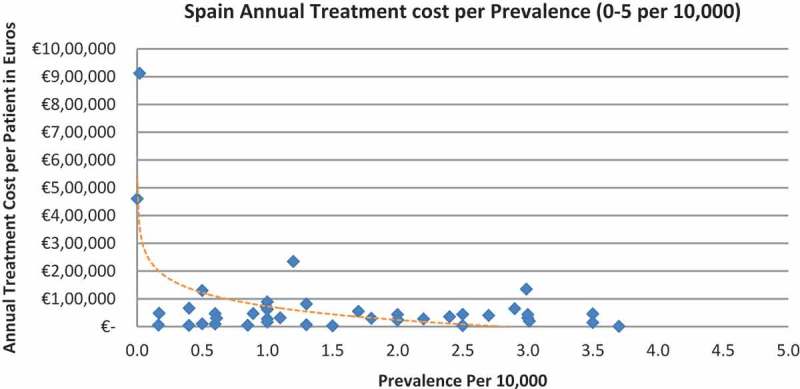
Spain annual treatment cost per prevalence (0–5 per 10,000).

**Figure 11.**
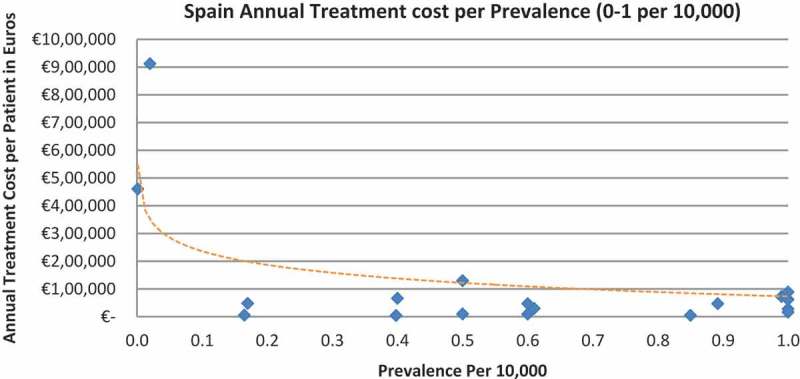
Spain annual treatment cost per prevalence (0–1 per 10,000).

**Figure 12.**
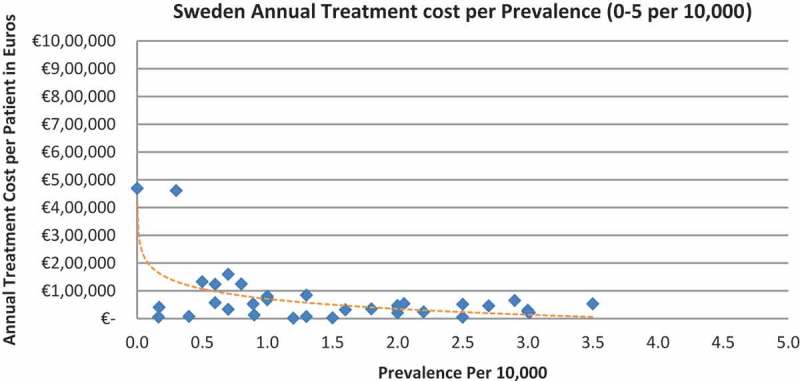
Sweden annual treatment cost per prevalence (0–5 per 10,000).

**Figure 13.**
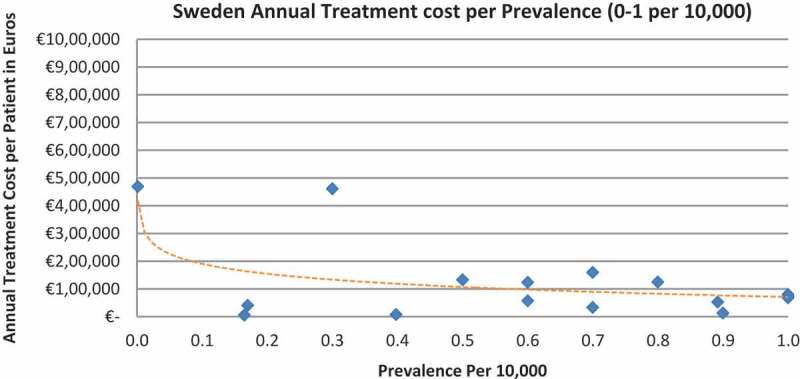
Sweden annual treatment cost per prevalence (0–1 per 10,000).

**Figure 14.**
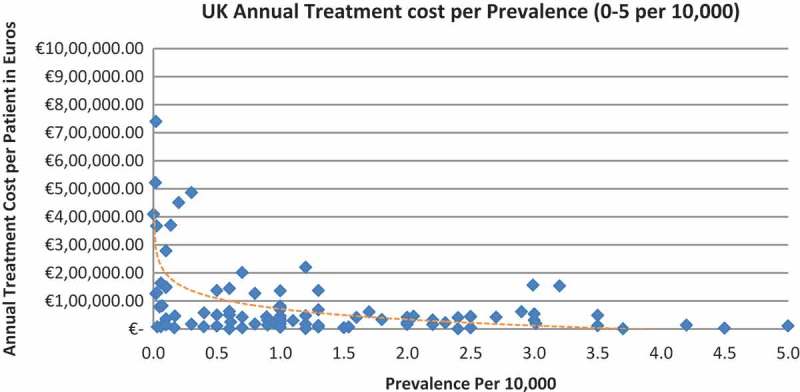
UK annual treatment cost per prevalence (0–5 per 10,000).

**Figure 15.**
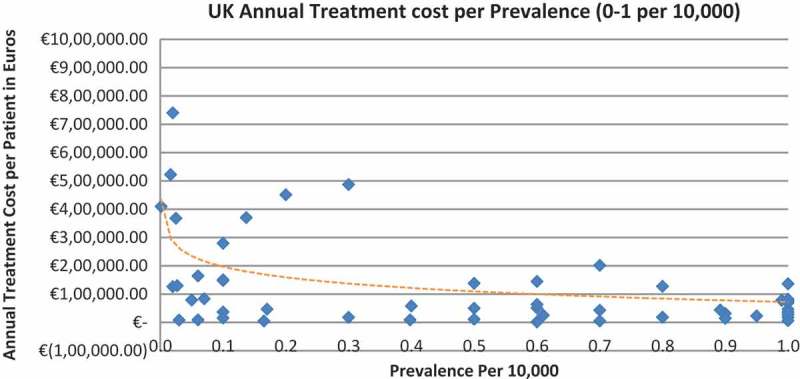
UK annual treatment cost per prevalence (0–1 per 10,000).

When we modelled the data where the logarithm of the prevalence was used as the exogenous variable, it showed an inverse linear relationship between prevalence and cost. Figures are presented in the supplementary file.

The sub analysis in France showed a statistically significant direct correlation between prevalence and ASMR, with a lower ASMR score (higher additional benefit) given the rarer the disease (r = 0.257, *p *< 0.05). It also showed a statistically significant inverse correlation between ASMR and the annual treatment costs, with the treatment with the lower ASMR score (higher additional benefit) being more expensive (r = −0.265, *p *< 0.05).

## Discussion

Publications related to predictors of price in rare diseases, specifically with rarity as an explanatory variable, are few (Table 3). Most of these studies are not comprehensive in terms of the number of orphan drugs analysed and the number of European countries scoped. These studies were also performed when fewer orphan drugs were launched. To our knowledge this work is the most comprehensive research assessing rarity as a determinant of price in orphan drugs to date.

**Table 3.** Available literature assessing the correlation between orphan drugs annual treatment costs and disease prevalence.

Publication	Year	Geographical scope	Number of orphan drugs analysed	Methodology	Results: correlation between annual treatment cost and disease prevalence
Alcimed [36]	2005	France	10	Public price including taxes (PPIT) used	Inverse
Messori et al. [37]	2010	Italy	17	Non-cancer orphan drugs included; Drug prices from Italian national drug regulatory agency used	Inverse
Aballéa et al. [38]	2010	France, Italy, Germany, Spain, UK	51	Public prices used	Inverse
Picavet et al. [14]	2014	Belgium, Czech Republic, France, Italy, the Netherlands, UK	59	Public prices used	Inverse
Onakpoya et al. [39]	2014	UK	74	Annual costs based on literature search	Inverse

This study shows a significant inverse relationship between orphan drug annual treatment cost and disease prevalence in Europe. Although sample size was not that large and despite price being a multifactorial outcome, statistical significance was met in all countries. These results are aligned with the results of all the previous studies presented in Table 3.[14,36–39]

The number of drugs analysed per country varied, with Sweden (N = 35) and Spain (N = 42) having the least drugs analysed due to the lower number of orphan drugs with publicly available prices. The low sample size in Sweden probably explains why the inverse correlation between annual costs and the rarest diseases prevalence (prevalence of 0–1 per 10,000) did not meet statistical significance. Only 16 drugs out of the 35 were analysed, compared to 19 to 52 drugs in the other countries when considering the lowest prevalence disorders.

Annual treatment costs were used to compare prices because it is not possible to compare prices directly from the database as it only provides prices per available pack, and thus adjustments to annual treatment prices had to be performed for the purpose of this analysis. The comparison of annual treatment costs of orphan drugs among countries shows that the costs of drugs in northern European countries are higher than in southern Europe. Sweden was again an outlier probably due the smaller sample size. Annual treatment costs ranges from as low as less than €1,000 to more than €1,000,000. This is a wide range considering that all of these drugs are designated to rare, life-threatening or chronically debilitating conditions. The cost range is the same for the rarest disease cohort (prevalence of 0–1 per 10,000). The drug with the lowest annual cost is Onsenal (celecoxib) for the treatment of familial adenomatous polyposis (FAP) with a disease prevalence of 0.3 per 10,000. Onsenal has an annual treatment cost per patient of €755 in Italy. It has however been withdrawn from the market by EMA post-approval at the request of the marketing authorisation holder (MAH) due to MAH’s inability to provide the additional data required, as a result of slow enrolment in an ongoing clinical trial.[40] The drug with the highest annual cost is Glybera (alipogene tiparvovec) for the treatment of familial lipoprotein lipase deficiency (LPLD) which has a prevalence of 0.02 per 10,000. It is the first gene therapy drug that has been approved by the EMA and is currently only available in Germany at a launch annual treatment cost of €1,051,956 per patient. In between these two extreme annual costs, the data range shows an inverse relationship between cost and rarity.

Pricing of orphan drugs is a complex process with multiple price determinants. Two recent studies looked into the decision drivers of orphan drugs price setting in Europe and both supported the general notion that orphan drug pricing in Europe is inconsistent and non-transparent. Onakpoya et al. [39] in 2014 showed that the annual cost of drugs in the UK did not appear to be related to their clinical effectiveness and that there is no clear and standardised mechanism for determining their prices. The difficulties in generating evidence for rare diseases and the lack of robust information when the price is set are probable factors. Picavet et al. [14] in 2014, through a multiple regression analysis, showed that prices of orphan drugs in six EU countries are influenced by factors such as the availability of an alternative drug treatment, repurposing of the drug, the length of treatment, the administration route, the presence of multiple indications, and the impact in overall survival and quality of life (QoL). The study however indicates that relevant vagueness still surrounds the orphan drug pricing mechanism.

In most countries, severity of disease has an impact on P&R decisions in Europe. This was exemplified by the NICE Citizen’s Council Report.[2] No studies were found analysing the correlation of rare disease severity to price. A reason for this is that there is no reliable data available for rare diseases on disease severity that can be used in a regression analysis.[14] It is worthwhile to investigate if disease rarity contributes to disease severity perception in payer value assessments. If rarity is found to contribute to severity and payers value severity, it can be deduced that payers value rarity. The sub analysis in France assessed the relationship between disease rarity and severity in payer assessments. The French P&R system uses two scores to assess a drug’s value: the Service Médical Rendu (SMR), also called the Actual Benefit (AB), and the Amélioration du Service Médical Rendu (ASMR), also called the Improvement in Actual Benefit (IAB). SMR is the driver of reimbursement decision and an insufficient SMR leads to non-reimbursement. SMR is based on several criteria such as disease severity, treatment efficacy and safety, the type of treatment (preventive, curative or symptomatic), its position in the therapeutic strategy, the presence of alternative treatment options and its impact on public health.[41] Severity being one of the criteria shows that payers in France value severity. On the other hand, ASMR is the driver for price determination and reflects drug efficacy compared to existing treatments.[41] ASMR has five levels ranging from one to five (I to V). A score of I signifies major improvement or a therapeutic breakthrough and a score of V signifies no clinical improvement. ASMR from I to III leads to a price premium. Our sub analysis showed a statistically significant direct correlation between prevalence and ASMR, where a lower ASMR score was given the rarer the disease. It also showed a significant inverse correlation between ASMR and the annual treatment costs, where higher costs were observed with lower ASMR scores. This sub analysis strengthens the results of our study that payers in France value rarity and is reflected in the lower ASMR scores and higher price premiums for rarer diseases. Payers in France also value severity through positive reimbursement decisions. Although this sub analysis has not shown that disease rarity contributes to disease severity, which in turn leads to a higher price due to the specificities in the French system, where disease severity is a driver of reimbursement and not price, this may still be a probable scenario in other countries with different P&R systems. At the same time, almost all orphan drugs were assessed with high SMR scores unlike non-rare conditions.

In the UK, the Citizen’s Council report [2] showed that willingness to pay is not driven by rarity and NICE HTA has been known to uphold cost per QALY benchmarks as the norm. However, our results showed otherwise and that payers in the UK valued rarity. A comprehensive study on the use of incremental cost per QALY gained in ultra-rare disorder by Schlander et al. [21] discussed that a growing body of literature considers cost per QALY economic evaluations in ultra-rare diseases as flawed, raising concerns on equity as these ultra-rare diseases are unlikely to meet standard cost per QALY benchmarks. The study argued that these traditional HTAs are an oversimplification of the complexities of health care priority setting and decision making, and that social values cannot be simply associated with decreasing incremental cost-effectiveness ratios (ICERs). The same study discussed that the introduction of the ‘ultra-orphan’ category by NICE as well as another category called ‘end-of-life treatments’ can be interpreted to be made in response to public pressure on NICE with regards to discussions around horizontal equity versus vertical equity, and the need for an evaluation framework which reflects social preferences while remaining consistent to normative commitments but addresses the limitation of the logic that all QALYs are created equal.[21] This can be interpreted that a differential cost per QALY benchmark may have been considered in the UK, valuing rarity in the evaluation of ultra-rare diseases. Therefore, one may consider that the NICE and SMC decision frameworks which favour horizontal equity through a predefined cost per QALY do not apply to orphan drugs in actuality. Ultra-rare diseases operate through a process that favours vertical equity. The ambiguity highlights the complexity in operating through a single framework for both rare and more common diseases.

All countries that opt for vertical equity, such as Germany, France and Sweden, as well as countries that opt for horizontal equity, such as the UK, value rarity in practice and tend to pay higher prices for the lowest prevalence. While this is consistent for the former group, it comes as a surprise for the latter.

Our methodology and results are in line with a previous study by Picavet et al. [14] which showed that orphan drug prices are determined based on the prevalence of the first indication. Launch prices for the first indication are unlikely to be reviewed following approval and combined prevalence is not a determinant for price setting, thus multiple indications for an orphan drug are associated with higher prices.[14]

Although drug price is a multivariate decision, this study answers the question: do payers value rarity? Payers in Europe do although it is not the sole criteria on which drug pricing is based on. The weight of the value given to rarity varies per health care system as well. This study sheds some understanding on the value drivers that payers attach to orphan drugs in light of the ongoing discussion of whether rarity should be valued and how European health care systems should assess and price orphan drugs. This study provides robust evidence that payers value rarity with a high correlation and that the correlation increases when tested on the most rare disease segment (prevalence of 0–1 per 10,000). Assessment of value of orphan drugs considers factors such as disease rarity and severity, unmet clinical need, clinical benefit and budget impact. The rarity of a disease does only minimally affect the cost of development and therefore rarity inevitably leads to a notably higher price point. A sustainable price level is essential in ensuring long term innovation for patients with rare diseases.

### Limitations of this research

The prevalence data at the EU level may not accurately represent the actual rate in the countries analysed. Rare disease prevalence often represents the number of clinically diagnosed patients, but more patients may be exposed to the disease. Another point to consider is that only a percentage of these patients may have clinically significant disease which warrants treatment, and not all patients are treated. However, although differences in prevalence may exist, these are expected to be infrequent phenomena.

In terms of treatment indications, only one indication per orphan drug was included in the analysis. This may have skewed the correlation as treatment costs of the other approved indications were not included. However, the first EMA-approved indications were chosen and the earliest prices were taken into account in the analysis and thus reflected pricing decisions upon launch.

Lastly, the prices of the drugs are listed prices which are often not aligned with the actual net prices, the latter being a better reflection of health care expenditures on orphan drugs. Confidential discounts, rebates, and tenders may be negotiated at the national, regional, or provider level, which may distort the ex-factory price. However, the potential discounts and rebates are expected to be reasonably homogeneous for all orphan drugs within the same country and prevalence will unlikely drive rebates or discounts during negotiations.

## Conclusion

In all the countries in scope, this study shows an inverse correlation between annual treatment cost and disease prevalence with high statistical significance. Although pricing is a complex process where different attributes are assessed, this study suggests that payers in all the countries value rarity of disease in pricing decisions. This analysis generated robust results which can support the continuous discussion on the development of more consistent and transparent value assessments of orphan drugs in Europe.

## Supplementary Material

Supplementary Data
