# Supplementary material for: Do payers value rarity? An analysis of the relationship between disease rarity and orphan drug prices in Europe
Source: J Mark Access Health Policy. 2017 Apr 10;5(1):1299665. doi: 10.1080/20016689.2017.1299665 (PMC5405566; doi:10.1080/20016689.2017.1299665)
Supplement: Supplementary Data [file zjma_a_1299665_sm6078.docx]

Supplementary Data

**Figure 16. France Annual Treatment cost per Log(prevalence per 10,000)**

**Figure 17. Germany Annual Treatment cost per Log(prevalence per 10,000)**

**Figure 18. Italy Annual Treatment cost per Log(prevalence per 10,000)**

**Figure 19. Spain Annual Treatment cost per Log(prevalence per 10,000)**

**Figure 20. UK Annual Treatment cost per Log(prevalence per 10,000)**

**Figure 21. Sweden Annual Treatment cost per Log(prevalence per 10,000)**

**Figure 22. Norway Annual Treatment cost per Log (prevalence per 10,000)**
